# Conservative Management of Intrascrotal Polyorchidism in a 14-Year-Old Boy: A Case Report and Review of the Current Literature

**DOI:** 10.1155/criu/5258413

**Published:** 2025-09-09

**Authors:** Panagiotis Nikolinakos, Abhisekh Chatterjee, Efstratios Christianakis, Ioannis Alexandrou, Nikolaos Chatzikrachtis, Elisavet Kotsi, Viktor Alargkof, Ivo Donkov, Samuel Bishara, Nikolaos Zavras, Joseph M. Norris

**Affiliations:** ^1^Department of Urology, West Middlesex University Hospital, Chelsea and Westminster Hospital NHS Foundation Trust, London, UK; ^2^Department of Pediatric Surgery, School of Medicine, Attikon University Hospital, National and Kapodistrian University of Athens, Athens, Greece; ^3^Imperial College School of Medicine, Imperial College London, London, UK; ^4^Department of Pediatric Surgery, Penteli Children's Hospital, Athens, Greece; ^5^Department of Pediatrics, Penteli Children's Hospital, Athens, Greece; ^6^Department of Urology, University Hospital Basel, Basel, Switzerland; ^7^UCL Division of Surgery & Interventional Science, University College London, London, UK

**Keywords:** paediatric surgery, polyorchidism, triorchidism, urological surgery, urology

## Abstract

Polyorchidism, or supernumerary testes (SNTs), is a rare congenital condition, management of which remains debated, particularly in paediatric cases with other concomitant features. We report a case of intrascrotal polyorchidism in a 14-year-old boy managed surgically due to parental preference and the need for histological confirmation. The patient presented with a 2-week history of painless heaviness in the scrotum. Physical examination and Doppler ultrasonography revealed a 1.8 cm mass fused to the inferior pole of the left testicle with associated Grade 1 varicocele, hydrocele and testicular appendix. Although MRI of the scrotum was initially offered, the family declined in favour of timely histological confirmation. Surgical exploration confirmed a fused supernumerary testicle and a biopsy showed normal spermatogenesis; this was consistent with Type A3 triorchidism. The patient had no complications or recurrence of symptoms at 12-month follow-up. This case highlights the use of surgical exploration in selected intrascrotal polyorchidism cases where imaging can be inconclusive or histological confirmation is required. Parental concerns and long-term reassurance may also reasonably influence management decisions.

## 1. Introduction

Polyorchidism is a rare congenital anomaly defined as the presence of three or more testes [1]. The incidence of polyorchidism is thought to be very low, as approximately only 200 cases have been published in the literature thus far [1]. Triorchidism is the most common form of this condition and typically presents asymptomatically or is discovered incidentally during childhood or early adolescence [2]. Depending on their location, supernumerary testes (SNTs) can exhibit an increased risk of malignancy or torsion [3]. Despite the development of evidence-based management frameworks for polyorchidism [2], the importance of concomitant features and individual patient characteristics often drive management decisions, which we explore further in a review of the most recent literature. Here, we present the case of a 14-year-old boy with histologically proven triorchidism and concomitant varicocele managed conservatively following surgical exploration.

## 2. Case Presentation

**Figure 1:**
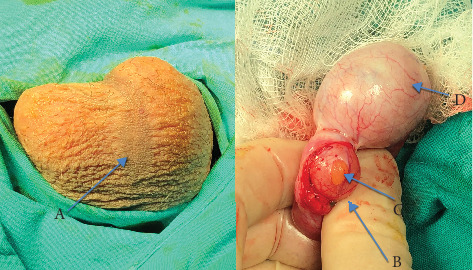
(A) Scrotum with wide raphe. (B) Supernumerary testis. (C) Parenchyma of the supernumerary testis. (D) Left testicle.

**Figure 2:**
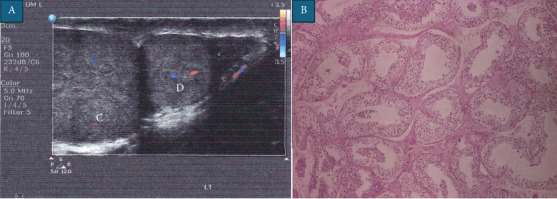
(A) Doppler ultrasonography of the scrotum and supernumerary testicle. (B) Haematoxylin and eosin stain of the biopsy of the supernumerary testicle (100×). (C) Left testicle. (D) Supernumerary testicle.

A 14-year-old male presented to our outpatient clinic with a 2-week history of a painless sense of heaviness and fullness in the scrotum. In the medical history, there were no reports of trauma, urinary tract infections, abdominal symptoms, evidence of dysuria, previous surgeries or congenital conditions. There was no family history of testicular masses or congenital malformations. On close inspection of the penis and scrotum, there were no abnormalities identified apart from a wider than usual scrotal raphe (Figure 1). Physical examination revealed a normal right testicle and normal cord. On palpation of the left hemiscrotum, a 1.5 cm mass, with consistency like an epididymal cyst, was noted. A full abdominal examination was unremarkable. Following this, a panel of relevant blood tests, including a full blood count (FBC), alpha-fetoprotein (AFP), human chorionic gonadotropin (hCG) and a lactate dehydrogenase (LDH), were all returned with normal results. On Doppler ultrasonography, the left and right testicles measured 3.64 × 2.62 and 4.84 × 2.14 cm, respectively. Moreover, a 1.8 × 1.5 cm mass fused with the left testicle at the inferior pole (Figure 2) was found. It exhibited similar echogenicity and vascularity as the testes, and a diagnosis of Type A3 polyorchidism was suggested. Taking into consideration the sonographical appearance, a differential diagnosis of a bilobed testicle could also have been made. Cohen et al. reported a paediatric case of bilateral undescended bilobed testes, and suggested conservative management for uncomplicated cases of polyorchidism; De Carli et al. presented a similar case of a bilobed testicle and offered a similar suggestion [4, 5]. As polyorchidism is an extremely rare presentation, the family was very keen on histological confirmation in order to conclusively exclude any sinister features despite the reassuringly normal echotexture of the testicle on sonography. A small testicular appendix was noted on the left side, together with a small hydrocele and a concomitant Grade 1 varicocele. Neither the hydrocele nor the varicocele had been palpable on examination. The right testicle and cord were normal. An MRI of the scrotum was offered but declined by the patient's parents due to fear of delaying the biopsy and subsequent histopathological analysis of the mass.

Under general anaesthesia, the left hemiscrotum was accessed with a transverse incision, and the layers were dissected down to the testis. The left testicular appendix was identified and diathermised. The supernumerary testis (SNT) was observed to share a vascular supply and vas deferens with the left testicle. A biopsy of the parenchyma of the SNT was taken (Figure 1). Histopathologic examination (Figure 2) revealed no abnormality and normal spermatogenesis. This confirmed the radiological and intraoperative clinical diagnosis of Type A3 polyorchidism, as defined by Bergholz et al. [3], or Type 2 polyorchidism as defined by Leung et al. [6]. Postoperatively, the patient's testosterone levels were found to be within normal range for his age. The patient's SNT had no change on examination or ultrasonographic appearance at last follow-up 12 months postoperatively. The patient reported that his symptoms of heaviness had subsided and subsequently did not wish to get the varicocele ligated at that time.

## 3. Discussion

Due to the rarity and often vastly heterogeneous presentations of polyorchidism, case reports such as this constitute the majority of the evidence-based literature on the topic. Cases of polyorchidism have been reported in the literature for more than 120 years [2]. The exact aetiology of polyorchidism is unknown. Longitudinal or transverse duplication of the genital ridge and primordial gonad with two ridges are possible pathways to formation of supernumerary testicles [1]. Most cases are asymptomatic and are discovered incidentally during imaging studies, physical examination or surgery for unrelated conditions [3].

Regarding the management of SNTs, two large meta-analyses have been published—one in 2009 [3] and another in 2023 [2]. Bergholz et al. [3] created a classification system for SNTs, whereas Balawender et al. [2] developed a simplified management algorithm. Bergholz et al. [3] identified cryptorchidism as being a major risk factor for malignancy and thus proposed that extrascrotal SNTs should be orchiectomised. Pliszka et al. have also suggested that nonscrotal SNTs should be removed due to the risk of malignancy, regardless of any potential impact on reproductive potential [1]. In line with this recommendation, Balawender et al. [2] found that only 41% of intrascrotal SNTs underwent surgical intervention compared to 92% of extrascrotal SNTs. When viewing our patient's SNT through the management algorithm alone [2], an MRI and referral for regular observation would have been sufficient. Polyorchidism is commonly classified by Bergholz et al. into types based on reproductive potential. Type A testes have an associated vas deferens and may be functional, whilst Type B do not. Our patient had Type A3 polyorchidism, where the SNT shares both the epididymis and vas deferens with the adjacent testis, supporting a conservative approach if malignancy is excluded [3].

However, the identification of a testicular appendix (torsion of which is often the causative agent of acute scrotal pain in prepubertal males [7]), the presence of a concomitant varicocele and the parents' wish to rule out malignancy were drivers in opting for surgical exploration rather than an MRI and subsequent observation; the latter may well have been an acceptable evidence-based approach. Surgical exploration allowed us to eliminate the future risk of torsion of the testicular appendage, examine the concomitant features of the SNT and confirm its benign nature histopathologically. However, surgical intervention does not come without its risks. The risk of testicular damage and resulting infertility in such a young patient had to be considered and communicated with the patient and their parents. Moreover, despite there being no evidence that unfused intrascrotal SNTs carry a greater risk of malignancy, Bergholz et al. [3] did postulate that these SNTs have a greater risk of intrascrotal torsion due to the absence of a gubernaculum testis (or scrotal ligament), a sentiment echoed by other authors [8]. Indeed, this had occurred in two of the cases [7, 9] we identified in our review of recently published (between 2022 and 2024) reports of polyorchidism within the literature (Table 1). In our patient, whose SNT was small, fused to the left testicle and not supplied by its own vasculature, an increased risk of torsion was unlikely.

Our review of recent case reports (Table 1) highlighted a broad range of concomitant pathologies, with varying management approaches [7, 9–17]. Two of these [10, 15] went against the recent suggestions by Balawender et al. [2], similarly to ours. However, cases of polyorchidism with a concomitant varicocele are rare, reportedly as low as 1.4% [3]. We only identified two cases in the literature of polyorchidism with concomitant varicocele [17, 18]. One was not ligated [17], whilst the other was [18], likely due to its higher grade, the presence of an unfused SNT of Grade 3A and oligoasthenozoospermia.

In conclusion, the management of polyorchidism can be complex. Each case must be considered individually; as our case highlights, management is patient and context specific. Especially within the paediatric population, factors such as the patients' age, proximity to pubertal onset and the wishes of their family must be carefully considered alongside the SNT's location and risk of malignancy. Trends in the management of SNTs can help guide decision-making processes but must not be solely relied upon, especially when concomitant features are present. Further primary research is needed to quantify the risk of malignancy in extrascrotal SNTs and the risk of torsion in both fused and unfused intrascrotal SNTs. In cases where the findings of scrotal ultrasonography are indeterminate, urologists should employ a high degree of clinical suspicion and promptly consider a subsequent MRI or scrotal exploration. Scrotal ultrasonography, whilst accessible and useful, can fail to provide a definitive diagnosis of scrotal pathology [19].

## Figures and Tables

**Table 1:** A summary of published cases of polyorchidism between January 2022 and September 2024 and their concomitant features and management.

**Citation**	**Age (years)**	**Number of SNTs**	**Location of SNTs**	**Concomitant features**	**Management**
Varun et al. [10]	40	1	Left inguinal canal	None	Observation
Li et al. [11]	6	2	Intrascrotal	None	Observation
Arena et al. [12]	7	1	Intrascrotal	Orthotropic duplication of the right testes with a preserved vascular supply following second stage of Fowler–Stephen orchidopexy of both testicles	Observation
Dirie et al. [13]	17	1	Intrascrotal	Accessory testis with normal sonography and function	Observation
Sarmiento et al. [14]	9	1	Right inguinal canal	Finding of two nonatrophic testes sharing a single epididymis and ductus deferens	Orchidopexy
Xiaofei et al. [7]	16	1	Intrascrotal	Testicular torsion of the SNT	Orchiectomy
Ojaghzadeh et al. [15]	25	3	2× inguinal canal, 1× left hemiscrotum	Patient refused biopsy	Observation
Nikolic et al. [16]	28	1	Distal spermatic cord	None	Orchiectomy
Kanbar et al. [17]	7	1	Intrascrotal	Grade IV varicocele on the left-hand side	Observation
Cardoso et al. [9]	17	2	Intrascrotal	Bilateral testicular torsion in bilateral polyorchidism	Orchiectomy and orchidopexy

## Data Availability

Data sharing is not applicable to this article as no datasets were generated or analysed during the current study.
